# Data on Tougu Xiaotong capsules may inhibit p38 MAPK pathway-mediated inflammation in vitro

**DOI:** 10.1016/j.dib.2019.105023

**Published:** 2019-12-19

**Authors:** Xihai Li, Zhenli Zhang, Wenna Liang, Jianwei Zeng, Xiang Shao, Limei Xu, Liangliang Jia, Xiaojuan He, Hui Li, Chunsong Zheng, Hongzhi Ye, Tetsuya Asakawa

**Affiliations:** a Academy of Integrative Medicine, Fujian University of Traditional Chinese Medicine, Fuzhou, 350122, PR China; b Fujian Key Laboratory of Integrative Medicine on Geriatrics, Fuzhou 350122, PR China; c SIPO Patent Examination (Beijing) Center, Beijing 100160, PR China; d Research Base of Traditional Chinese Medicine Syndrome, Fujian University of Traditional Chinese Medicine, Fuzhou 350122, China; e Department of Neurosurgery, Hamamatsu University School of Medicine, Handayama, 1-20-1, Higashi-ku, Hamamatsu-city, Shizuoka 431-3192, Japan

**Keywords:** Osteoarthritis, Tougu Xiaotong capsule, Inflammation, Chondrocyte, Toll like receptor 4

## Abstract

The Tougu Xiaotong capsule (TXC) is a traditional herbal compound used to treat osteoarthritis (OA) in China. We performed fingerprint analysis with HPLC for the quality control of TXC. Its composition was identified by the comparison of the spectrogram and chromatographic peak of retention time with a reference substance. TXC was found to contain paeoniflorin, isofraxidin, ferulic acid, and rosmarinic acid. The chondrocytes were identified by immunohistochemical staining using collagen II. Chondrocytes that were positive for collagen II were stained brown in the cytoplasm. The toll-like receptor 4 (TLR4) was expressed on the chondrocyte membrane, which was observed using immunofluorescence microscopy. The nuclei were stained blue by 4′,6-diamidino-2-phenylindole (DAPI) and TLR4 was stained green. These were observed using laser scanning confocal microscopy. The successful establishment of LPS-exposed chondrocytes was confirmed using enzyme-linked immunosorbent assay (ELISA). Lipopolysaccharide (LPS) administration significantly reduced the levels of interleukin-1β (IL-1β) and tumor necrosis factor-α (TNF-α), and a maximum effect was observed at 8 h. We believe that these methods will be useful in future investigations of OA. This data article is related to the research article “Tougu Xiaotong capsules may inhibit p38 MAPK pathway-mediated inflammation: *In vivo* and *in vitro* verification” [1].

**Table:** Specifications Table

Subject	Medicine and Dentistry
Specific subject area	Investigation of the mechanisms of Tougu Xiaotong capsule in treating osteoarthritis, an orthopaedics disease
Type of data	Figures
How data were acquired	Fingerprint analysis was performed via a high-performance liquid chromatography (HPLC) fingerprint method using an Agilent 1200 HPLC system (Agilent, Santa Clara, CA, USA). Type II collagen immunohistochemical staining was observed with a light microscope (BH2; Olympus, Tokyo, Japan). TLR4 immunofluorescence was checked with a laser scanning confocal microscope (LSM710; Zeiss, German). ELISA kits (R&D Systems, USA) were used for confirming enhancement of IL-1β and TNF-α in culture solution exposed to LPS.
Data format	Raw and analyzed
Parameters for data collection	Ultimate™ XB-C18 column (4.60 × 250.00 mm, 5 μm, Welch Materials, Inc., USA) were used for HPLC. Chondrocytes were cultured in low-glucose DMEM (Gibco, USA) supplemented with 10% fetal calf serum (Gibco), penicillin (100 U/mL), and streptomycin (100 μg/mL). Chondrocytes were exposed to 10 ng/mL LPS (Sigma-Aldrich, USA) for 8 h to establish the cellular model. Cells were exposed to 10 ng/mL LPS for 4, 8, 12, or 24 h.
Description of data collection	We assessed the quality of TXC by fingerprint analysis using HPLC with an Agilent 1200 HPLC system. The identified components of TXC followed previous studies, and the HPLC data confirmed the high quality of the TXC extract. Chondrocytes were cultured in low-glucose DMEM (Gibco, USA) supplemented with 10% fetal calf serum (Gibco), penicillin (100 U/mL), and streptomycin (100 μg/mL). Passage 2 chondrocytes were identified by immunohistochemical analysis using collagen II (Col II). The expression of toll-like receptor (TLR) 4 on chondrocytes was detected using immunofluorescence microscopy. To establish a cell model, chondrocytes were exposed to 10 ng/mL LPS (Sigma-Aldrich, USA) for 4, 8, 12, and 24 h. We measured the levels of IL-1β and TNF-α in the cell supernatants collected from cultured cells because the anti-inflammatory effects of IL-1β and TNF-α occurred only when they were secreted into the cell matrix. The increased levels of IL-1β and TNF-α in the culture medium indicated that we had succeeded in establishing a LPS-exposed cellular model. The maximum effect was observed at 8 h. Therefore, we selected an 8-h exposure for subsequent experiments.
Data source location	Institution: Fujian University of Traditional Chinese Medicine City/Town/Region: Fuzhou Country: China
Data accessibility	All data were included in the submitted manuscript. The raw data are provided as a supplementary file.
Related research article	Xihai Li, Zhenli Zhang, Wenna Liang, Jianwei Zeng, Xiang Shao, Limei Xu, Liangliang Jia, Xiaojuan He, Hui Li, Chunsong Zheng, Hongzhi Ye*, Tetsuya Asakawa*, Tougu Xiaotong capsules may inhibit p38 MAPK pathway-mediated inflammation: in vivo and in vitro verification, Journal of Ethnopharmacology, 10.1016/j.jep.2019.112390

**Table:** 

**Value of the Data** • This study performed a quality control for herbal compounds. We have provided simple and objective methods for determining the main active ingredients and performing quality control of herbal compounds. • These data demonstrate that chondrocytes can be identified by evaluating the expression of TLR4. This can be used as a standard method to identify chondrocytes in the study of orthopedic diseases. • In this study, we measured the levels of pro-inflammatory cytokines, such as IL-1β and TNF-α, in cell supernatants. This method can be used to confirm the negative effects of a certain toxicant. Moreover, by identifying the maximum and minimum effects of the toxicant, we can select the most appropriate conditions (such as dose and duration) for its exposure.

## 1 Data description

**Fig. 1:**
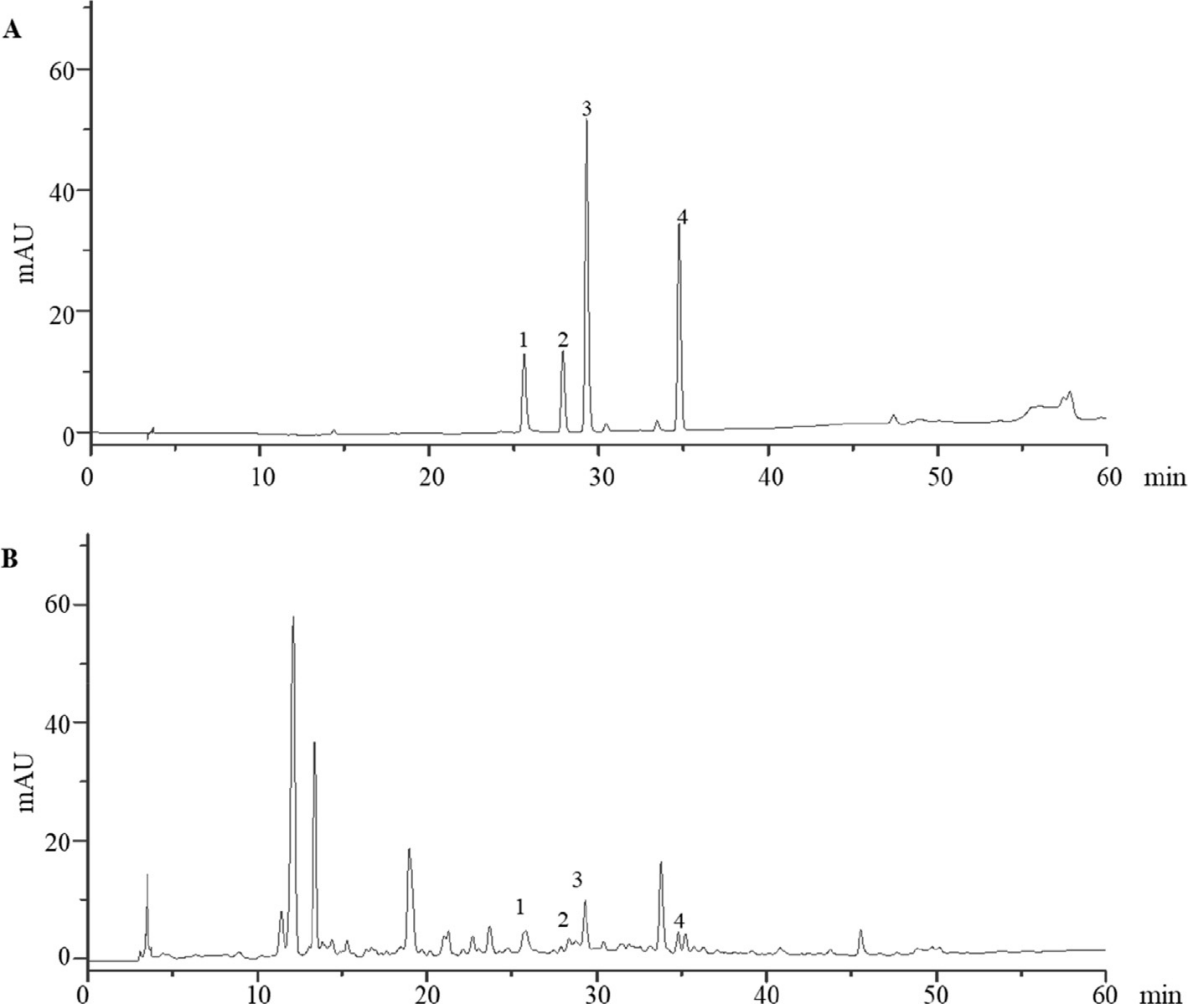
Data of the fingerprint analysis of TXC using HPLC. A. Liquid chromatogram of the reference substance. B. Liquid chromatogram of TXC. Both the reference substance and TXC exhibited four peaks, namely paeoniforin (1), isofraxidin (2), ferulic acid (3), and rosmarinic acid (4).

The quality control data are shown in Fig. 1. Fig. 1A shows the liquid chromatogram of the reference substance, paeoniflorin, isofraxidin, ferulic acid, and rosmarinic acid. These four chromatographic peaks (Fig. 1B) indicate the good quality of the TXC solutions used in our experiments. The raw data used to generate Fig. 1 are provided in section 1 of the supplementary file.

**Fig. 2:**
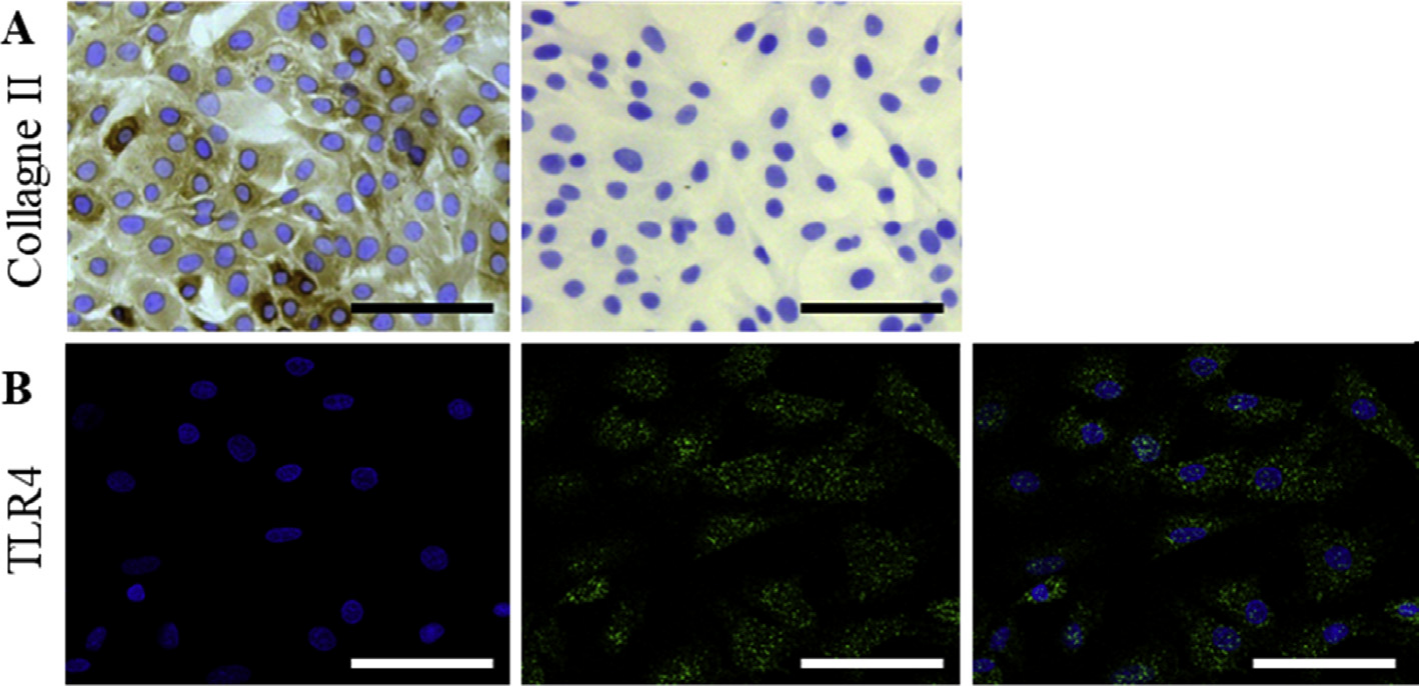
Identification of the chondrocytes. A. Chondrocytes were identified by collagen II immunohistochemistry. Cells positive for collagen II (chondrocytes) were stained brown in the cytoplasm (left). B. Chondrocytes were examined by immunofluorescence. Nuclei were stained blue by DAP and TLR4 was stained green observed by using laser scanning confocal microscopy. Bars = 200 μm.

Fig. 2 shows the representative micrographs of the chondrocytes. The collagen II immunohistochemistry data is shown in Fig. 2A. Many of the cells were found to be collagen II-positive (brown) using immunofluorescence. These chondrocytes were selected for the next experiments. Fig. 2B shows the immunofluorescence data of TLR4. DAPI staining revealed blue nuclei and green TLR4 when observed using a laser scanning confocal microscope. LPS was used to establish the cellular model of inflammation. The raw data used to generate Fig. 2 are provided in section 2 of the supplementary file.

**Fig. 3:**
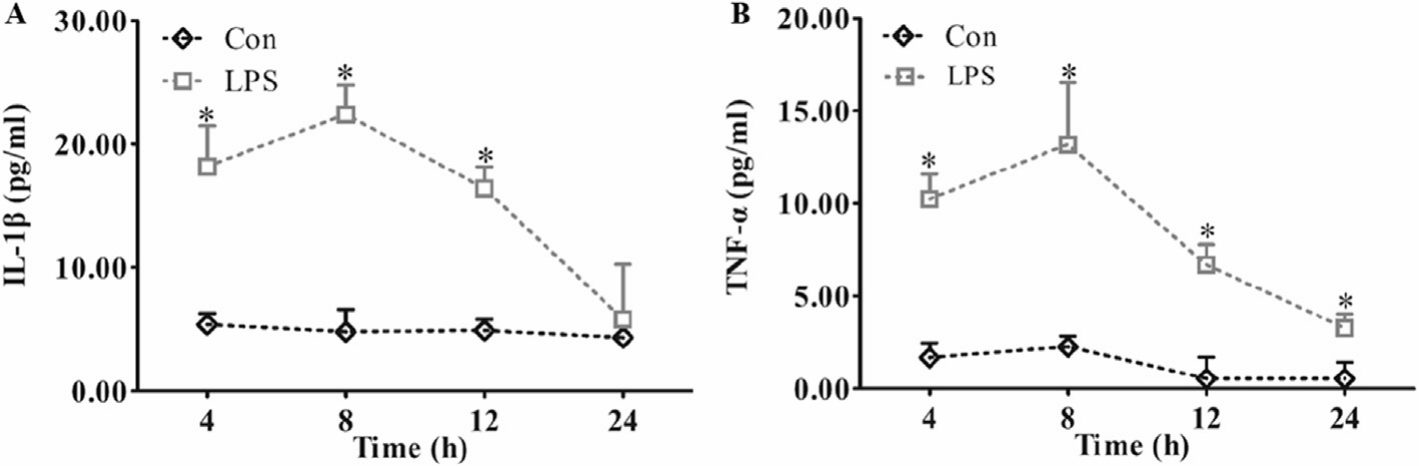
Confirmation of successful establishment of LPS-exposed chondrocytes. LPS administration significantly reduced IL-1β (A) and TNF-α (B) levels. Administration at 8 h achieved the maximum effect. *p < 0.05; **p < 0.01.

Fig. 3 shows the changes in IL-1β and TNF-α levels in cell supernatants after the cells were exposed to 10 ng/mL LPS for 4, 8, 12, and 24 h. The data of IL-1β (Fig. 3A) and TNF-α (Fig. 3B) were analogous. LPS increased the levels of IL-1β and TNF-α over time, and the maximum effect occurred at 8 h. We confirmed the successful establishment of LPS-exposed chondrocytes and then selected the 8-h exposure time of LPS for the following experiments. The raw data used to generate Fig. 3 are included in section 3 of the supplementary file.

## 2 Experimental design, materials, and methods

### 2.1 TXC extracts and fingerprint analysis

TXC herbs were prepared using the methods described in our previous studies [2]. Briefly, we extracted 108 g of herbal powder and dissolved it in 1.5 L distilled water refluxing twice for 2 h each. The TXC filtrate was evaporated with a rotary evaporator (RE-2000; Shanghai Yarong Biochemical Instrument Factory, Shanghai, China) and dried to a constant weight using a vacuum drying oven (DZF-300; Shanghai Hengke Electronic Technology Co., Ltd., Shanghai, China). The quality control of TXC extracts was performed using an Ultimate™ XB-C18 column (4.60 × 250.00 mm, 5 μm, Welch Materials, Inc., USA). The HPLC fingerprint assay was performed using an Agilent 1200 HPLC system (Agilent, Santa Clara, CA, USA) under the following conditions: a mobile phase of methanol-0.1% phosphoric acid, detection wavelength of 277 nm, flow rate of 1 mL/min, column temperature of 30 °C, gradient procedure 5% A at 0–5 min, 5%–20% A at 5–10 min, 20%–42% A at 15–25 min, 42%–65% A at 25–40 min, 65%–80% A at 40–55 min, and 80%–100% A at 55–70 min. The standard substances used were paeoniflorin, isofraxidin, ferulic acid, and rosmarinic acid (National Institute for Pharmaceutical and Biological Products Control, Beijing, China) [2].

### 2.2 Collagen II immunohistochemistry assay

We isolated chondrocytes from the knee articular cartilage of male Sprague-Dawley rats that were 4 weeks old (Shanghai Slack Laboratory Animal Co., Shanghai, China). As described in our previous study, passage 2 cells were identified using a collagen II immunohistochemical staining assay [3]. Briefly, sterilized coverslips were placed in the wells of a 24-well plate. Subsequently, we seeded the chondrocytes at a density of 2 × 10^5^ cells/well. The chondrocytes were cultured in an incubator that was maintained at 5% CO_2_ and 37 °C for 48 h. Immunohistochemical staining was performed using a primary antibody against collagen II (1:100; ab34712, Abcam) and an immunoglobulin IgG secondary antibody (1:2000; ab97051, Abcam). Images were recorded using a light microscope (BH2; Olympus, Tokyo, Japan) [1].

### 2.3 TLR4 immunofluorescence assay

Cells were placed in a laser confocal dish that was fixed with 4% formaldehyde at 4 °C for 30 min, and incubated in 0.5% Triton at room temperature for 10 min. A primary antibody against TLR4 (1:200; ab22048, Abcam) and a secondary antibody (goat anti-rabbit IgG, H + L, 1:1000; A11008, Thermo Fisher Scientific, USA) were used in the immunofluorescence assay. Images were recorded using a confocal microscope (LSM710; Zeiss, German).

### 2.4 ELISA analysis

We used ELISA kits (R&D Systems, USA) to measure IL-1β and TNF-α levels in cell supernatants after treatment. We measured the levels in 100 μL samples by using a microplate spectrophotometer (Omega Bio-Tek, Inc., Norcross, GA, USA) at 405 nm [4].
