# Supplementary material for: Data on Tougu Xiaotong capsules may inhibit p38 MAPK pathway-mediated inflammation in vitro
Source: Data Brief. 2019 Dec 19;28:105023. doi: 10.1016/j.dib.2019.105023 (PMC6939087; doi:10.1016/j.dib.2019.105023)

**Raw Data**

**Section 1. Raw data of Figure 1**

**Liquid chromatogram of the reference substance**

**Liquid chromatogram of TXC**

**Section 2. Raw data of Figure 2**

**Collagen II immunohistochemistry**


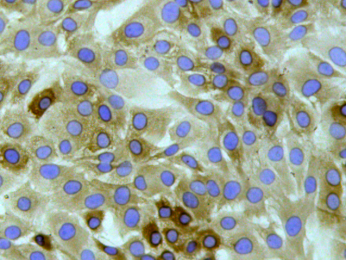

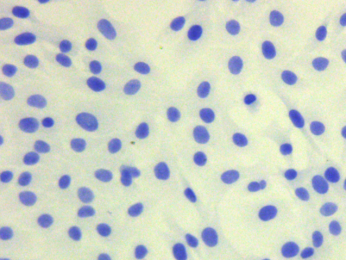


**TLR4 immunofluorescence**


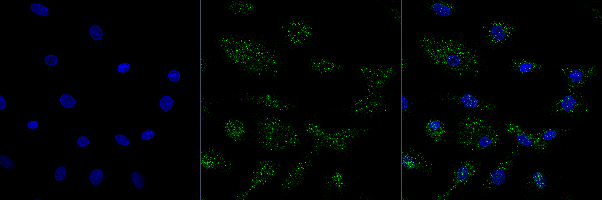


**Section 3. Raw data of Figure 3, levels of cytokines**

**IL-1β**

| Group | Time | IL-1β (pg/mL) | | | |
| --- | --- | --- | --- | --- | --- |
| Control | 4H | 3.757281 | 5.609465 | 4.220029 | 7.929172 |
|  | 8H | 2.832384 | 3.294733 | 2.832384 | 10.25386 |
|  | 12H | 4.220029 | 3.294733 | 4.682975 | 7.464833 |
|  | 24H | 2.832384 | 3.757281 | 3.294733 | 7.464833 |
| LPS | 4H | 18.66392 | 15.38568 | 17.25777 | 21.48161 |
|  | 8H | 21.95192 | 20.54158 | 22.42243 | 24.77797 |
|  | 12H | 15.38568 | 16.78945 | 15.38568 | 18.19500 |
|  | 24H | 6.073008 | 2.832384 | 4.220029 | 10.25386 |

| OD Value | Concentration |
| --- | --- |
| 0 | 0 |
| 0.1004 | 31.3 |
| 0.1257 | 62.5 |
| 0.2351 | 125 |
| 0.4587 | 250 |
| 0.8791 | 500 |
| 1.6311 | 1000 |
| 2.7182 | 2000 |


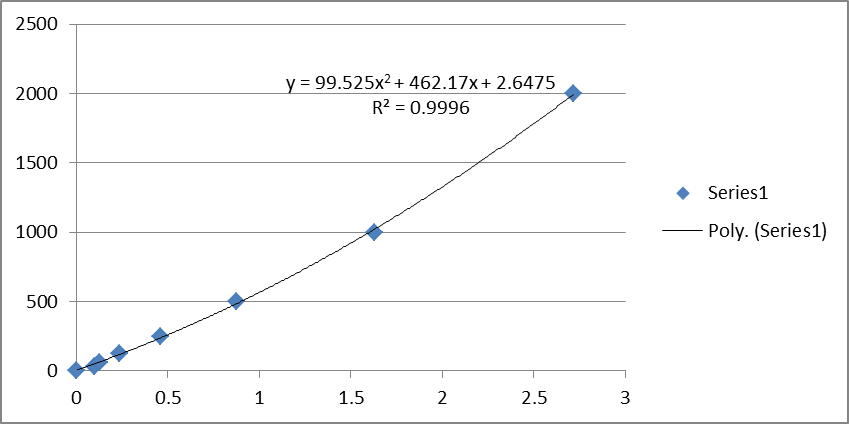


**TNF-α**

| Group | Time | TNF-α (pg/mL) | | | |
| --- | --- | --- | --- | --- | --- |
| Control | 4H | 1.878592 | 1.878592 | 1.499782 | 1.752322 |
|  | 8H | 1.499782 | 2.636607 | 2.257533 | 2.131307 |
|  | 12H | 0.742557 | 0.364142 | 1.499782 | 0.868827 |
|  | 24H | 0.742557 | 1.121104 | 0.364142 | 0.742601 |
| LPS | 4H | 8.719689 | 12.92136 | 9.101001 | 10.24735 |
|  | 8H | 9.101001 | 19.8312 | 10.62757 | 13.18659 |
|  | 12H | 8.719689 | 4.913812 | 6.434582 | 6.689361 |
|  | 24H | 3.774617 | 2.257533 | 3.774617 | 3.268922 |

| OD value | Concentration |
| --- | --- |
| 0 | 0 |
| 0.0564 | 12.5 |
| 0.0787 | 25 |
| 0.1357 | 50 |
| 0.2818 | 100 |
| 0.4729 | 200 |
| 0.9411 | 400 |
| 1.6557 | 800 |


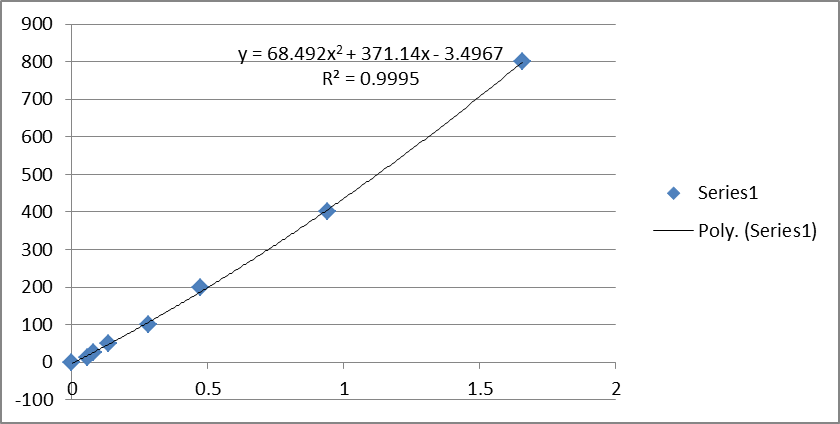

Supplement: Multimedia component 1 [file mmc1.docx]
